# Determinants of Poor Outcomes in Acute Poisoning at a Tertiary Care Hospital in an Industrial City of Eastern India: A Retrospective Observational Study

**DOI:** 10.7759/cureus.111840

**Published:** 2026-06-30

**Authors:** Murtaza A Khan, Saurabh Pathak, Md. Khalid Jung Khan, Nibedita Mishra

**Affiliations:** 1 Internal Medicine, Tata Main Hospital, Jamshedpur, IND; 2 Internal Medicine, Manipal-Tata Medical College, Jamshedpur, IND; 3 Nephrology, Tata Main Hospital, Jamshedpur, IND; 4 General Medicine, Tata Main Hospital, Jamshedpur, IND

**Keywords:** acute poisoning, aluminium phosphide, corrosive ingestion, drug overdose, industrial eastern india, jharkhand, logistic regression, organophosphate, poor outcome predictors, roc analysis

## Abstract

Background: Industrial cities of eastern India combine dense urban populations with proximity to agricultural hinterlands and access to pharmaceutical, household, and agricultural toxic agents, creating a mixed exposure profile. Published data from such settings remain sparse. This study characterised the clinical profile of acute poisoning at an industrial-urban tertiary care centre and evaluated factors associated with escalation of care.

Methods: This retrospective observational study included 322 eligible poisoning admissions identified from departmental records at a tertiary care centre in industrial eastern India between January 2023 and March 2025. The prespecified composite endpoint comprised intensive care unit (ICU) admission, mechanical ventilation, vasopressor support, or in-hospital death. Because all patients meeting the composite endpoint were admitted to the ICU, the endpoint was numerically equivalent to ICU admission in this cohort. Multivariable logistic regression and receiver operating characteristic (ROC) analyses were used to evaluate associated factors and assess exploratory discrimination.

Results: Median age was 29 years (IQR 21-39; mean 31.6 ± 13.7 years); 182 (56.5%) were female. Suicidal intent was documented in 151 patients (46.9%). Drug overdose (n=100, 31.1%; comprising 99 pharmaceutical overdoses and one alcohol-ingestion case) and corrosive ingestion (n=85, 26.4%) were the leading agent categories. A composite poor outcome was identified in 114 (35.4%) patients. Six patients died, giving an in-hospital mortality of 1.9% (6/322). On multivariable regression, lower Glasgow Coma Scale (GCS) score (odds ratio (OR) 2.73 per unit decrease, 95% confidence interval (CI) 1.64-4.55, p<0.001) and lower peripheral oxygen saturation (SpO₂; OR 1.14 per unit decrease, 95% CI 1.05-1.24, p=0.002) were independently associated with the composite endpoint, which was numerically equivalent to ICU admission in this cohort. The shock index had the largest point-estimate area under the ROC curve (area under the curve (AUC) 0.751, 95% CI 0.693-0.811), followed by heart rate (AUC 0.736) and GCS score (AUC 0.708); no formal pairwise comparison of AUCs was performed.

Conclusion: In this single-centre cohort, pharmaceutical overdose and household chemical ingestion were the most frequently recorded poisoning categories, alongside pesticide, rodenticide, and occupational chemical exposures. Lower admission GCS and SpO₂ were associated with the institution-dependent escalation-of-care endpoint. These findings should be considered hypothesis-generating and require validation using objective clinical outcomes in prospective multicentre cohorts.

## Introduction

Acute poisoning is a leading cause of emergency hospital admissions and preventable mortality worldwide, with the highest burden concentrated in low- and middle-income countries (LMICs). In India, poisoning accounts for 5-15% of all emergency admissions at tertiary care hospitals and approximately 60,000-80,000 deaths annually [1,2]. The epidemiology of poisoning is not uniform; it is profoundly shaped by socioeconomic context, occupational environment, geographic proximity to toxic agents, and cultural practices within a given community [3].

Industrial cities in eastern India represent an undercharacterised setting for poisoning research. The Jharkhand-Odisha-West Bengal corridor hosts some of the largest integrated steel plants, iron ore and coal mines, and chemical processing facilities in Asia, creating a complex demographic mix of organised-sector industrial workers, unorganised migrant labour, peri-urban communities, and adjacent tribal and rural populations [4]. This industrial-agrarian interface may result in a combination of pharmaceutical, household, agricultural, and occupational exposures within the same hospital catchment.

Several features of eastern India's industrial cities are of toxicological relevance. First, industrial and occupational exposures formed a small but locally relevant component of the cohort, accounting for 3.4% of admissions. These included exposures such as carbon monoxide (CO) and industrial solvents. Workplace conditions, occupational stress, and their relationship with poisoning intent were not evaluated in the present study; occupational context is therefore presented descriptively rather than as a causal explanation [5]. Second, the proximity of industrial cities to agricultural hinterlands sustains ready community access to organophosphate (OP) pesticides, rodenticides (including zinc phosphide and aluminium phosphide (ALP)), and plant toxins accessible in adjoining rural areas [6]. Third, pharmaceutical agents, particularly anxiolytics and analgesics, were prominently represented among intentional self-poisoning cases in the present cohort; benzodiazepines, particularly alprazolam, were a frequently encountered subclass in this context [7].

The existing Indian literature on acute poisoning originates predominantly from agricultural South India, metropolitan tertiary centres in Delhi or Mumbai, or general rural hospitals in Rajasthan and Uttar Pradesh [8-10]. Published reports specifically examining poisoning in industrial-urban populations of eastern India remain limited. This evidence gap limits the ability of hospital administrators, emergency physicians, and public health authorities in these cities to develop context-appropriate prevention and management strategies.

This study presents a comprehensive retrospective analysis of 322 acute poisoning admissions at a tertiary care referral hospital in an industrial city of eastern India. The study was designed to characterise the clinical and epidemiological profile of acute poisoning in this setting, which involves overlapping exposures to urban pharmaceuticals, household corrosives, agricultural pesticides, and occupational chemicals. The study also evaluated factors associated with the prespecified escalation-of-care endpoint using multivariable logistic regression and explored the discriminatory performance of selected admission variables using receiver operating characteristic (ROC) analysis. The composite endpoint was selected to capture clinically important deterioration requiring escalation of care; because all composite events in this cohort included ICU admission, its interpretation is closely linked to institutional ICU-triage practices.

## Materials and methods

Study design and setting

This was a retrospective observational study. All data were collected retrospectively from standardised case record forms (CRFs) maintained during routine clinical care at the Department of Medicine, Tata Main Hospital, Jamshedpur, Jharkhand, India, a tertiary-care teaching hospital in an industrial city of eastern India. The hospital is a multispecialty referral centre serving urban, peri-urban, and referred populations from Jamshedpur and the surrounding districts of eastern Jharkhand. It receives acute medical emergencies from within the city, as well as secondary referrals from peripheral hospitals and industrial occupational health units in the region. The requirement for individual informed consent was waived because the study involved retrospective review of existing clinical records. Data were handled confidentially; identifying information was excluded from analyses, manuscript tables, and all reported results. The study was conducted and reported in accordance with the Strengthening the Reporting of Observational Studies in Epidemiology (STROBE) guidelines for retrospective observational studies. The 13 core laboratory parameters were complete for all 322 patients in the analytic dataset; the GCS score was missing in four patients (1.2%). The completeness of the core dataset reflects the institution's standardised admission workup protocol and should not be interpreted as evidence of prospective data collection. All eligible poisoning records identified from the departmental case-record source during the study period were reviewed. The study may not have captured patients admitted exclusively under other departments or services unless their records were included in this source. Data were extracted from case record forms by a trained investigator using a pre-specified variable list; a 10% random sample was independently re-abstracted by a second investigator, and discordant values were resolved by consensus review.

Study duration

The study period was January 2023 to March 2025 (27 months). All 322 cases in the analytic dataset were admitted during this defined period and were identified retrospectively through systematic review of CRFs maintained by the Department of Medicine. Data from outside this study window were not included.

Inclusion and exclusion criteria

Patients were included if all of the following criteria were satisfied: (1) admitted to the Department of Medicine with a primary clinical diagnosis of acute poisoning confirmed by a treating physician on the basis of consistent history (witnessed ingestion, patient disclosure, or circumstantial evidence), clinical presentation, and/or toxicological findings; (2) age ≥12 years at the time of admission; (3) complete registration in the hospital medical records system with a unique Medical Records (MR) number; and (4) availability of a minimum dataset comprising age, gender, poison category, and clinical outcome. Patients were excluded for any of the following: chronic or cumulative poisoning without a discrete acute event; poisoning diagnosed incidentally during admission for an unrelated primary condition; transfer to another facility immediately after initial stabilisation without documented inpatient assessment at this center; duplicate MR records (first record retained); or records comprising only administrative identifiers with no clinical data. The complete inclusion and exclusion criteria are summarised in Table 1.

**Table 1:** Inclusion and exclusion criteria for study case selection MR = Medical Records. The source file contained 323 populated patient records; one exact duplicate (identified by matching MR number with identical clinical entries) was removed, leaving 322 unique records for analysis.

#	Criterion	Definition/Rationale
Inclusion 1	Primary diagnosis of acute poisoning	Diagnosis recorded by the treating physician on the basis of a compatible history, clinical presentation, and/or toxicological findings.
Inclusion 2	Age at least 12 years	Patients younger than 12 years are managed by the Department of Paediatrics.
Inclusion 3	Complete hospital registration	A unique medical record number allowed retrieval of the clinical record.
Inclusion 4	Minimum dataset	Age, gender, poison category, and final hospital outcome were available.
Exclusion 1	Chronic or cumulative toxicity	No discrete acute exposure event.
Exclusion 2	Incidental poisoning diagnosis	Poisoning was not the principal reason for admission.
Exclusion 3	Immediate transfer without inpatient assessment	No documented inpatient evaluation or treatment at the study centre.
Exclusion 4	Duplicate record	An identical duplicate registration was removed.
Exclusion 5	Identifier-only row	Administrative identifiers without clinical data.

Data cleaning and case flow

All admissions with a primary diagnosis of acute poisoning during the study period were identified. Case record forms were retrieved and systematically reviewed. One exact duplicate registration number was identified and removed. Eleven patients had a recorded hospital stay of zero days, representing same-calendar-day admissions; these values are retained as zero in all analyses. Stays were not imputed or recoded. Two cases with intent recorded as unknown were identified as keyboard-entry errors on CRF review and corrected to their verified classification. The final analytic cohort comprised 322 unique patient records admitted between January 2023 and March 2025.

Data variables

Variables extracted and analysed from the standardised CRFs included: (i) sociodemographic data: age (years), gender, and residence (urban/rural); (ii) risk factors: history of previous psychiatric illness, prior self-harm attempt, alcohol co-ingestion or regular use, and comorbidities; (iii) poisoning characteristics: agent category, specific agent name, access or source of the poison (including industrial and occupational sources), intent of poisoning (suicidal/accidental/unknown), route of exposure (oral/inhalational), and timing of presentation; (iv) clinical presentation: GCS score on admission (classified as severe when GCS <8, moderate at 8-13, and mild/normal when >13), vital signs at admission (systolic and diastolic blood pressure (BP), heart rate (HR), respiratory rate (RR), peripheral SpO₂), pupil findings (miosis, mydriasis, or normal), and presenting symptoms (vomiting, respiratory distress, shock, fasciculations, seizures, aspiration); (v) investigations: electrocardiogram (ECG) findings (normal, sinus tachycardia, QT prolongation, arrhythmia, ST-T changes) and chest X-ray (CXR) findings; (vi) interventions: gastric lavage (GL), activated charcoal (AC), atropine (total dose), pralidoxime (oxime therapy), vasopressor support, mechanical ventilation, intensive care unit (ICU) admission and duration (days), upper gastrointestinal endoscopy, and psychiatric referral; (vii) complications: aspiration pneumonia, acute respiratory distress syndrome (ARDS), acute kidney injury (AKI), arrhythmia, and GI injury/perforation/stricture. Complications were abstracted as documented by the treating physician in the case record form; uniform retrospective adjudication against formal diagnostic criteria was not performed; (viii) clinician-assigned severity grade (mild/moderate/severe); (ix) a composite poor outcome variable defined as ICU admission, mechanical ventilation, vasopressor support, or in-hospital death (any one criterion sufficient), defined a priori and independent of the clinician-assigned severity grade; these four components were selected because they collectively represent clinically significant deterioration requiring active intervention. In this cohort, all 114 patients meeting the composite endpoint were admitted to the ICU; no patient met the ventilation, vasopressor, or mortality component without also receiving ICU admission, so the composite endpoint is numerically equivalent to ICU admission in this cohort and should be interpreted in light of institutional ICU-triage practices; and (x) final clinical outcome, classified as discharged, discharged against medical advice (DAMA), or in-hospital death. Poisoning intent was classified by the treating physician based on patient or witness disclosure; cases without determinable intent were categorised as unknown. A local clinician-assigned severity category was recorded as mild, moderate, or severe, reflecting the treating physician’s assessment at admission. These categories were not equivalent to the standard International Programme on Chemical Safety/European Association of Poisons Centres and Clinical Toxicologists (IPCS/EAPCCT) Poisoning Severity Score. Because severity was assigned by the treating clinician using the same clinical information that subsequently determined ICU admission and organ-support decisions, comparisons of vital signs and interventions across severity categories are partly circular. The combined alcohol-exposure variable was coded positive when the CRF documented either acute alcohol co-ingestion or regular/habitual alcohol use at the time of poisoning.

Sample size

No formal a priori sample size calculation was performed, as this was a retrospective analysis of all eligible admissions within the defined study period. The regression subset comprised 318 patients and 113 composite-outcome events across six predictor variables, corresponding to an events-per-variable (EPV) ratio of 18.8, exceeding the recommended minimum of 10. Four patients with missing GCS scores were excluded from the model; one of these patients had experienced the composite outcome (accounting for the difference between 113 events in the regression subset and 114 events in the full cohort).

Statistical analysis

Continuous variables were assessed for normality using the Shapiro-Wilk test; given non-normal distributions, results are presented as the median with interquartile range (IQR) where appropriate, or mean ± standard deviation (SD) for normally distributed variables. Categorical variables are reported as frequency (n) and percentage. Between-group comparisons used the Mann-Whitney U test for continuous variables and Chi-square or Fisher's exact test for categorical variables, as appropriate. For comparisons of continuous variables across three severity grades (mild, moderate, severe), the Kruskal-Wallis test was used, with post hoc Dunn's test and Bonferroni correction. The composite poor outcome (ICU admission, mechanical ventilation, vasopressor support, or in-hospital death) was defined a priori. Shock was recorded by the treating clinician at presentation. Among 41 patients recorded as shocked, 24 had systolic BP <90 mmHg and 39 had a Shock Index (HR/SBP) ≥1.0; two clinically classified cases did not meet either threshold, reflecting that clinical shock assessment incorporated features beyond these numerical criteria alone. Shock on admission was excluded from the multivariable model because of complete separation (all 41 shocked patients experienced poor outcomes), which precludes valid standard maximum-likelihood estimation; shock is reported univariately using Fisher's exact test. GCS and SpO₂ were entered as continuous covariates to avoid complete separation with their binary cutpoints. Variance inflation factor (VIF) was calculated to assess multicollinearity. Corrected VIFs (calculated with model intercept) were: GCS score 1.37, SpO₂ 1.36, Age 1.07, Male sex 1.07, organophosphate (OP) poisoning 1.04, alcohol co-ingestion 1.02. These values indicate no meaningful multicollinearity between any predictors in the model. For regression analyses, male sex was coded as 1; female, transgender, and other categories were coded as 0. Four patients with missing GCS scores were excluded from the regression model, giving an analytic N of 318. 

Model calibration was assessed with the Hosmer-Lemeshow test; a non-significant result (HL χ²=4.30, df=8, p=0.829) indicated no evidence of lack of fit. Odds ratios (OR) with 95% confidence intervals (CI) are reported. The final multivariable model was limited to six variables selected on the basis of clinical relevance, avoidance of redundant physiological measures, and model parsimony. Systolic blood pressure, heart rate, creatinine, and bicarbonate, although significant on univariate comparison (all p<0.001), were not included in the final model and were evaluated separately in exploratory ROC analyses as continuous bedside markers. Shock on admission was excluded because of complete separation. Discriminatory ability of continuous predictors was evaluated using ROC analysis; AUC with 95% CI from 1,000 bootstrap iterations (seed=42) is reported for individual predictors. For the multivariable model, the AUC confidence interval (0.712-0.824) was also obtained by 1,000 bootstrap iterations of model predictions (seed=42); this reflects apparent in-sample performance, not optimism-corrected discrimination. Optimal cutpoints were identified using the Youden index; these are data-derived thresholds requiring external validation before clinical application. Missing data were minimal: GCS was missing for 4 of 322 patients (1.2%), all of whom were excluded from analyses requiring GCS; all other core variables had complete data. No imputation was performed. Statistical significance was defined as p<0.05 (two-tailed). Because ICU admission and organ-support interventions formed part of the composite endpoint, some predictors may partially reflect clinician-driven escalation pathways (incorporation bias); OR estimates should be interpreted accordingly. All analyses were performed using Python 3.12 (scipy 1.11, statsmodels 0.14, scikit-learn 1.3, pandas 2.0; www.python.org). Figures were generated using matplotlib 3.7 at 300 DPI (https://matplotlib.org/).

## Results

Sociodemographic profile and risk factors

A total of 322 unique acute poisoning cases were analysed. The median age was 29 years (IQR 21-39; mean 31.6 ± 13.7 years; range 12-80). Young adults aged 18-30 years constituted the largest group (n=136, 42.2%), followed by those aged 31-45 years (n=95, 29.5%). Adolescents aged 12-17 years accounted for 42 cases (13.0%); patients younger than 12 years were excluded. Females comprised n=182 (56.5%) of admissions and males n=137 (42.5%). Urban residents accounted for n=289 cases (89.8%); the remaining n=33 cases (10.2%) were referred from surrounding rural districts. History of previous psychiatric illness was documented in n=58 patients (18.0%), recorded as a dedicated risk-factor variable separate from physical comorbidities; prior self-harm attempt was documented in 38 patients (11.8%). Alcohol co-ingestion or habitual use was documented in 59 patients (18.3%). Physical comorbidities were present in 39 patients (12.1%), most commonly hypertension, diabetes mellitus, and chronic obstructive pulmonary disease (COPD)/asthma; psychiatric illness was captured separately as a risk-factor variable. Psychiatric referral was advised in n=125 cases (38.8%), encompassing 82.8% of all suicidal admissions (n=125/151). The sociodemographic and risk factor data are presented in Table 2.

**Table 2:** Sociodemographic profile, risk factors, and intent of poisoning (N=322) IQR = interquartile range (25th–75th percentile); SD = standard deviation. Proportions are calculated with N=322 as the denominator unless otherwise stated. The Other category comprises two patients documented as transgender and one patient recorded as Other; these are combined because of the small cell size. †The accidental category comprises 82 accidental exposures and one recreational exposure.

Characteristic	n (%)	Notes
Age		
Mean ± SD (years)	31.6 ± 13.7	Range 12–80
Median (IQR)	29 (21–39)	-
<18 years	42 (13.0%)	-
18–30 years	136 (42.2%)	-
31–45 years	95 (29.5%)	-
46–60 years	34 (10.6%)	-
>60 years	15 (4.7%)	-
Gender		
Female	182 (56.5%)	-
Male	137 (42.5%)	-
Others	3 (0.9%)	Two transgender; One recorded as Other
Residence		
Urban	289 (89.8%)	Urban catchment area
Rural	33 (10.2%)	
Time to Presentation-Mean (hours)	3.2	Range 0.3–12.4
Risk Factors		
Previous psychiatric illness	58 (18.0%)	
Previous self-harm attempt	38 (11.8%)	
Alcohol co-ingestion / habitual use	59 (18.3%)	36 female; 21 male; 2 transgender
Comorbidity present	39 (12.1%)	Hypertension, diabetes mellitus, and COPD/asthma are the most frequent among physical comorbidities; psychiatric illness was captured as a separate risk-factor variable (n=58) and is excluded from this count
Intent of Poisoning		
Suicidal	151 (46.9%)	
Accidental	83 (25.8%)	Includes occupational, domestic, and recreational (n=1) exposure†
Unknown/undetermined	88 (27.3%)	-
Psychiatric referral-suicidal cases	125/151 (82.8%)	% of 151 suicidal admissions
Psychiatric referral-all cases	125/322 (38.8%)	0 referrals in accidental or unknown-intent cases

Poisoning agent distribution and source

**Figure 1:**
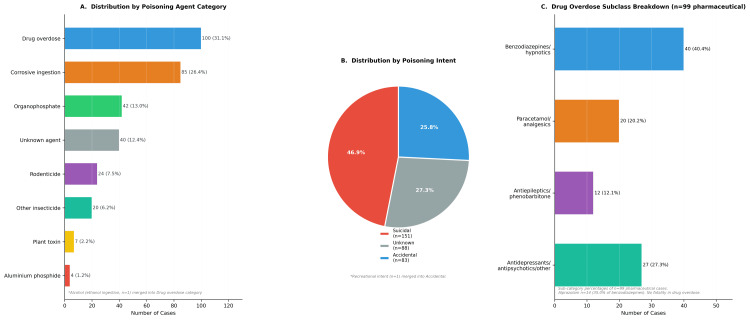
Agent category distribution, intent of poisoning, and drug overdose subclass breakdown (N=322) The drug-overdose category total of 100 comprises 99 pharmaceutical overdoses and one alcohol-ingestion case; the accidental-intent category comprises 82 accidental exposures and one recreational exposure. (A) Horizontal bar chart showing distribution of admissions by agent category. Drug overdose (n=100, 31.1%) and corrosive ingestion (n=85, 26.4%) together accounted for 57.5% of admissions. Three of the four patients with ALP poisoning died; this descriptive finding is based on a very small subgroup. Values shown as count and percentage of N=322. (B) Pie chart showing distribution by poisoning intent: suicidal n=151 (46.9%), unknown/undetermined n=88 (27.3%), accidental n=83 (25.8%). Among suicidal cases, 82.8% (n=125/151) received a formal psychiatric referral recommendation. (C) Horizontal bar chart of drug overdose subclasses (n=99). Benzodiazepines/hypnotics were the largest subclass (n=40, 40.4%); alprazolam (n=14) was the most common single agent. No fatality occurred in the drug overdose category. OP = organophosphate; ALP = aluminium phosphide; COR = corrosive; ROD = rodenticide; OI = other insecticide; TCA = tricyclic antidepressant.

Drug overdose was the most frequent agent category (n=100, 31.1%), followed by corrosive ingestion (n=85, 26.4%), OP poisoning (n=42, 13.0%), and unknown agents (n=40, 12.4%). Rodenticides accounted for n=24 cases (7.5%) and other insecticides for n=20 (6.2%); ALP was identified in n=4 cases (1.2%) (Figure 1). Notably, n=11 cases (3.4%) were attributed to industrial chemicals or occupational exposures - a source category rarely reported in Indian poisoning literature outside industrial settings. Suicidal intent predominated (n=151, 46.9%), with accidental exposure in n=83 cases (25.8%) and unknown intent in n=88 (27.3%) (Figure 1). The oral route accounted for n=313 cases (97.2%); clinically suspected inhalational poisoning was documented in 9 cases (2.8%), involving carbon monoxide (n=4), organic solvent or diesel vapour (n=2), acid fumes (n=1), aerosol insecticide (n=1), and smoke exposure (n=1); diagnoses were based on clinical presentation and exposure history as formal toxicological confirmation was not available.

Within the drug overdose group (n=99 pharmaceutical cases), benzodiazepines and related hypnotics constituted the largest subclass (n=40, 40.4%), with alprazolam (n=14) the single most common agent, followed by clonazepam (n=7) and zolpidem (n=2). Paracetamol was identified in n=20 cases (20.2%). Among corrosive cases (n=85), phenyl-based household floor cleaners dominated (n=62, 72.9%), including Phenyl, white phenyl, phenol variants, and Lysol. Chlorpyrifos-cypermethrin combination products were the predominant OP agents (n=13, 31.0%). The specific agent distribution is presented in Table 3.

**Table 3:** Specific poisoning agents by category and frequency (N=322) The drug-overdose category total of 100 comprises 99 pharmaceutical overdoses and one alcohol-ingestion case; subclass percentages use 99 as the denominator. OP = organophosphate; COR = corrosive; ALP = aluminium phosphide; TCA = tricyclic antidepressant; ICU = intensive care unit. Agent categories are mutually exclusive; the primary or most clinically significant agent was used for classification where co-ingestion occurred. Industrial/occupational exposures are distributed across corrosive, unknown agent, and other categories based on the substance involved.

Agent Category	n	% of Total	Key Agents and Notes
Drug overdose (Drug)	100	31.1%	Benzodiazepines/hypnotics n=40 (40.4%); alprazolam n=14, clonazepam n=7; paracetamol/analgesics n=20 (20.2%); antiepileptics n=12; no fatality in this category
-Benzodiazepines/hypnotics	40	40.4% of DRUG	Alprazolam n=14 (35.0%); clonazepam n=7; zolpidem n=2
-Paracetamol/analgesics	20	20.2% of DRUG	Paracetamol-containing cases n=20 (15 paracetamol alone; 5 mixed paracetamol-containing ingestions)
-Antiepileptics/phenobarbitone	12	12.1% of DRUG	Phenobarbitone predominant
-Antidepressants/antipsychotics/other	27	27.3% of DRUG	TCA, risperidone, trihexyphenidyl, pregabalin, antihistamines
Corrosive ingestion (COR)	85	26.4%	Phenyl-based floor cleaners n=62 (72.9%); acid n=8; alkali n=6; other n=9
-Phenyl/Lysol (household cleaners)	62	72.9% of COR	Most common corrosive agent in this cohort
Organophosphate pesticide (OP)	42	13.0%	Chlorpyrifos-cypermethrin n=13 (31.0%); chlorpyrifos n=8; unknown OP n=21
Unknown/unidentified agent	40	12.4%	Agent not identifiable from history, witness account, or initial investigations
Rodenticide	24	7.5%	Zinc phosphide n=11; other/unknown rodenticide n=13
Other insecticide (non-OP)	20	6.2%	Pyrethroid and related household insecticides predominated; individual agents included cypermethrin, carbamate, and other or unidentified products.
Plant toxin	7	2.2%	Datura, Nerium oleander, Calotropis; 1 inhalational (cannabis)
Aluminium phosphide (ALP)	4	1.2%	Three severe and one moderate case; all required ICU admission; three required ventilation; three died
Route of exposure			
Oral ingestion	313	97.2%	Predominant route across all categories
Inhalational	9	2.8%	Carbon monoxide n=4; organic solvent or diesel vapour n=2; acid fumes n=1; aerosol insecticide n=1; cannabis/smoke exposure n=1

Clinical presentation and severity

**Figure 2:**
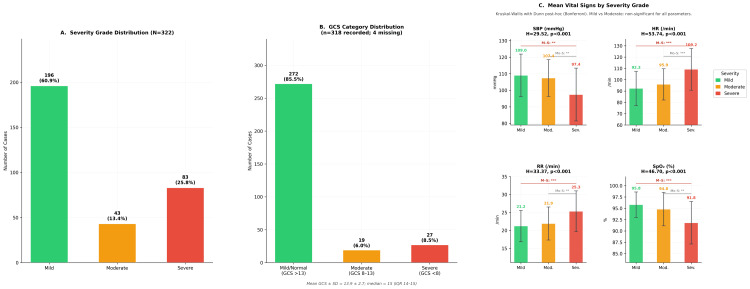
Severity grade distribution, GCS category distribution, and mean vital signs stratified by severity grade (N=322) (A) Bar chart showing severity grade distribution: Mild n=196 (60.9%), Moderate n=43 (13.4%), Severe n=83 (25.8%). Severity was graded using a local clinician-assigned category (mild/moderate/severe) by the treating physician. (B) Bar chart of GCS categories on admission (n=318 recorded; 4 missing): Severe (GCS <8) n=27 (8.5%); Moderate (8–13) n=19 (6.0%); Mild/Normal (>13) n=272 (85.5%). Percentages are of 318 recorded cases. Mean GCS ± SD = 13.9 ± 2.7; median = 15 (IQR 14–15). (C) Grouped bar chart showing mean values of SBP (mmHg), HR (/min), RR (/min), and SpO₂ (%) by severity grade. Kruskal-Wallis testing confirmed significant differences across all three severity grades (SBP: H=29.52, p<0.001; HR: H=53.74, p<0.001; RR: H=33.37, p<0.001; SpO₂: H=46.70, p<0.001). Post-hoc Dunn’s test (Bonferroni): no significant differences between mild and moderate grades; separation driven by the severe grade group (all p<0.01 vs mild and moderate). Error bars = ±1 SD. Overall means: SBP 105.8 ± 14.4 mmHg; HR 97.2 ± 17.4 /min; RR 22.4 ± 5.1 /min; SpO₂ 94.6 ± 3.9%. GCS = Glasgow Coma Scale; SBP = systolic blood pressure; HR = heart rate; RR = respiratory rate; SpO₂ = peripheral oxygen saturation; SD = standard deviation.

The mean GCS on admission was 13.9 ± 2.7 (median 15, IQR 14-15; range 1-15). Of 318 patients with a recorded GCS score (4 missing, 1.2%), severe GCS depression (<8) was present in n=27 cases (8.5%), moderate impairment (8-13) in n=19 (6.0%), and mild/normal GCS (>13) in n=272 (85.5%). Mean SpO₂ on admission was 94.6 ± 3.9%; n=93 patients (28.9%) presented with SpO₂ below 94%, and n=36 (11.2%) below 90%. Mean time to hospital arrival was 3.2 hours (range 0.3-12.4 hours). Vomiting was the most common presenting symptom (n=88, 27.3%), followed by respiratory distress (n=69, 21.4%), shock on admission (n=41, 12.7%), fasciculations (n=27, 8.4%), seizures (n=25, 7.8%), and suspected aspiration (n=22, 6.8%). Miosis was documented in 57 patients (17.7%), most frequently in OP poisoning, whereas mydriasis was recorded in 47 patients (14.6%) across several agent categories. Normal pupils were documented in 218 patients (67.7%). Severity grading revealed n=196 mild (60.9%), n=43 moderate (13.4%), and n=83 severe (25.8%) cases. A composite poor outcome was identified in n=114 patients (35.4%). Severe-grade patients demonstrated markedly lower mean systolic BP (97.4 vs 109.0 mmHg), higher heart rate (109.2 vs 92.3/min), and lower SpO₂ (91.8 vs 95.8%) compared to mild-grade cases (Figure 2). The full clinical presentation profile is presented in Table 4.

**Table 4:** Clinical presentation on admission (N=322) Values are frequency n (%) unless otherwise stated. GCS was available for 318 patients; GCS-category percentages use 318 as the denominator. The composite endpoint comprised ICU admission, mechanical ventilation, vasopressor support, or in-hospital death and was numerically equivalent to ICU admission in this cohort. Severity was recorded using a local clinician-assigned mild, moderate, or severe category and was not equivalent to the standard International Programme on Chemical Safety/European Association of Poisons Centres and Clinical Toxicologists (IPCS/EAPCCT) Poisoning Severity Score. BP = blood pressure; SpO₂ = peripheral oxygen saturation; GCS = Glasgow Coma Scale; IQR = interquartile range; SD = standard deviation; ICU = intensive care unit.

Parameter	Overall (N=322)
Vital signs on admission	
Systolic BP, mean ± SD (mmHg)	105.8 ± 14.4
Diastolic BP, mean ± SD (mmHg)	68.7 ± 9.1
Heart rate, mean ± SD (/min)	97.2 ± 17.4
Respiratory rate, mean ± SD (/min)	22.4 ± 5.1
SpO₂, mean ± SD (%)	94.6 ± 3.9
SpO₂ <90%	36 (11.2%)
SpO₂ <94%	93 (28.9%)
Glasgow Coma Scale (GCS)	
GCS, mean ± SD	13.9 ± 2.7
GCS, median (IQR)	15 (14–15)
GCS <8	27/318 (8.5%)
GCS 8–13	19/318 (6.0%)
GCS >13	272/318 (85.5%)
Pupil findings	
Miosis	57 (17.7%)
Mydriasis	47 (14.6%)
Normal pupils	218 (67.7%)
Presenting symptoms	
Vomiting	88 (27.3%)
Respiratory distress	69 (21.4%)
Shock on admission	41 (12.7%)
Fasciculations	27 (8.4%)
Seizures	25 (7.8%)
Suspected aspiration	22 (6.8%)
Local clinician-assigned severity category	
Mild	196 (60.9%)
Moderate	43 (13.4%)
Severe	83 (25.8%)
Composite endpoint	114 (35.4%)

Clinical interventions

Gastric lavage was performed in n=145 patients (45.0%) and activated charcoal was administered in n=92 (28.6%). Atropine was administered in 39 patients (12.1%), all of whom were classified as having OP poisoning. Pralidoxime (oxime therapy) was used in n=28 cases (8.7%), exclusively in OP poisoning. Vasopressor support was required in n=34 patients (10.6%). Mechanical ventilation was provided in n=53 cases (16.5%) and ICU admission in n=114 (35.4%), with a mean ICU stay of 3.4 days (maximum 23 days). Upper gastrointestinal endoscopy was performed in 58 of 85 patients with corrosive ingestion (68.2%); no endoscopies were performed in other agent categories. Zargar grade ≥IIa mucosal injury was documented in 41 of the 58 patients who underwent endoscopy (70.7%), corresponding to 41 of 85 patients in the overall corrosive-ingestion group (48.2%). Because endoscopy was performed selectively, the 41/85 estimate should not be interpreted as the prevalence of mucosal injury among all patients with corrosive ingestion. Psychiatric referral was documented as advised in n=125 patients (38.8%), comprising 82.8% of all suicidal intent admissions (n=125/151). The complete intervention profile, stratified by severity grade, is presented in Table 5.

**Table 5:** Clinical interventions and management utilization by severity grade (N=322) ICU = intensive care unit; OP = organophosphate; COR = corrosive; UNK = unknown agent; GL = gastric lavage; AC = activated charcoal; GI = gastrointestinal. The composite poor outcome endpoint (ICU admission OR mechanical ventilation OR vasopressor support OR in-hospital death) was met by n=114 patients (35.4%).  Severity-stratified hospital stay n values reflect patients with available stay data within each severity grade.

Intervention / Measure	Total n (%)	Mild (n=196)	Moderate (n=43)	Severe (n=83)	Notes
Gastrointestinal decontamination					
Gastric lavage (GL)	145 (45.0%)	95 (48.5%)	18 (41.9%)	32 (38.6%)	Mean arrival 3.2 h; agent-specific timing was not analysed
Activated charcoal (AC)	92 (28.6%)	57 (29.1%)	10 (23.3%)	25 (30.1%)	Single dose predominantly
Specific Antidotes					
Atropine	39 (12.1%)	17 (8.7%)	6 (14.0%)	16 (19.3%)	OP only; mean dose 40.7 mg (range 2.0–92.9)
Pralidoxime (oxime therapy)	28 (8.7%)	14 (7.1%)	3 (7.0%)	11 (13.3%)	OP only; 66.7% of all OP cases
Organ support and intensive care					
ICU admission	114 (35.4%)	0 (0%)	31 (72.1%)	83 (100%)	Mean stay 3.4 days (range 1–23)
Mechanical ventilation	53 (16.5%)	0 (0%)	0 (0%)	53 (63.9%)	Invasive ventilation; all in severe grade
Vasopressor support	34 (10.6%)	0 (0%)	0 (0%)	34 (41.0%)	Norepinephrine or dopamine
Procedures and other interventions					
Upper gastrointestinal endoscopy	58 (18.0%)	34 (17.3%)	12 (27.9%)	12 (14.5%)	All in corrosive ingestion; modified Zargar grading applied
Psychiatric referral advised	125 (38.8%)	83 (42.3%)	16 (37.2%)	26 (31.3%)	82.8% of 151 suicidal cases; none documented in accidental/unknown cases
Hospital stay duration					
Overall, mean ± SD (days)	2.8 ± 3.1	—	—	—	n=318 with recorded hospital-stay duration
Mild severity	—	1.8 ± 1.0 (median 2)	—	—	n=195
Moderate severity	—	—	4.1 ± 3.2 (median 3)	—	n=42
Severe severity	—	—	—	4.4 ± 4.9 (median 3)	n=81

In-hospital complications and laboratory profile

**Figure 3:**
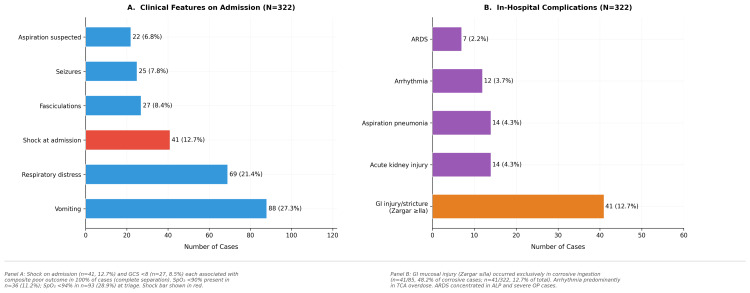
Presenting clinical features on admission and in-hospital complications (N=322) (A) Horizontal bar chart showing frequency of key clinical features at presentation. Vomiting (n=88, 27.3%) and respiratory distress (n=69, 21.4%) were the most common presenting features. Shock on admission (n=41, 12.7%) and GCS <8 (n=27, 8.5%) were each associated with a composite poor outcome rate of 100% (complete separation). SpO₂ <90% was present in n=36 (11.2%) and SpO₂ <94% in n=93 (28.9%) at triage. Values shown as n (%) of N=322. (B) Horizontal bar chart showing in-hospital complication rates. GI mucosal injury (Zargar ≥IIa) occurred exclusively in corrosive ingestion cases (n=41/85, 48.2% of corrosive cases; n=41/322, 12.7% of total). Aspiration pneumonia n=14 (4.3%), AKI n=14 (4.3%), arrhythmia n=12 (3.7%), ARDS n=7 (2.2%). GI = Gastrointestinal; Zargar IIa = Superficial focal ulceration (modified Zargar endoscopic grading); OP = Organophosphate; ALP = Aluminium phosphide; AKI = Acute kidney injury; ARDS = Acute respiratory distress syndrome; SpO₂ = Peripheral oxygen saturation; GCS = Glasgow Coma Scale.

Endoscopically documented GI mucosal injury (Zargar grade ≥IIa) was the most frequent complication (n=41, 12.7%), occurring exclusively in corrosive cases (n=41/85, 48.2%). Aspiration pneumonia and AKI each affected n=14 patients (4.3%). Arrhythmia was documented in n=12 cases (3.7%), and ARDS in n=7 (2.2%). ECG abnormalities were present in n=99 cases (30.7%), with sinus tachycardia the most common finding. Serum cholinesterase was available in 62 patients (including all 42 OP cases) but was not included in full-cohort analyses as it was not routinely measured across all poisoning categories. Patients with poor outcomes had significantly higher total leucocyte count (TLC; median 10,071 vs 9,153/µL, p<0.001), random blood sugar (RBS; 151 vs 136 mg/dL, p<0.001), creatinine (1.3 vs 0.9 mg/dL, p<0.001), lower median pH (7.33 vs 7.37, p<0.001), and lower bicarbonate (20.3 vs 22.7 mEq/L, p<0.001). Among the four ALP cases, the median urea was 60 mg/dL and the median pH was 7.25; these descriptive values should be interpreted cautiously because of the very small subgroup size. The complication rates and laboratory parameters, stratified by the composite endpoint, are presented in Table 6. The presenting clinical features and complication profile are illustrated in Figure 3.

**Table 6:** In-hospital complications and laboratory parameters by composite outcome (N=322) Composite endpoint = ICU admission OR mechanical ventilation OR vasopressor support OR in-hospital death (n=114, 35.4%). All 114 patients meeting the endpoint were admitted to the ICU; the endpoint is numerically equivalent to ICU admission in this cohort. † Complications were abstracted as documented by the treating physician in the CRF; uniform retrospective adjudication against formal diagnostic criteria was not performed. Categorical comparisons used Fisher’s exact test (two-tailed). Significant p-values (p<0.05) are shown in bold red. ‡ GI mucosal injury occurred exclusively in corrosive-ingestion patients. Upper gastrointestinal endoscopy was performed selectively in 58 of 85 corrosive-ingestion cases; 41 of the 58 scoped patients had Zargar grade ≥IIa injury (70.7%), corresponding to 41/85 (48.2%) of the overall corrosive group. Denominators for the poor/not-poor split reflect corrosive-ingestion patients within each outcome group (28 with, 57 without composite endpoint); GI injury p-value uses the full 322-patient denominator. § pH specimen source (arterial, venous, or capillary) was not consistently documented in the CRF and cannot be confirmed retrospectively. Serum cholinesterase is not included because it was not routinely measured. GI = gastrointestinal; AKI = acute kidney injury; ARDS = acute respiratory distress syndrome; AST = aspartate aminotransferase; ALT = alanine aminotransferase; INR = International Normalised Ratio; IQR = interquartile range (25th–75th percentile); ICU = intensive care unit; CRF = case record form; OP = organophosphate, PT = Prothrombin.

Parameter	Overall (N=322)	Composite Endpoint (n=114, 35.4%)	No Composite Endpoint (n=208, 64.6%)	p
Complications - n (%) (Fisher’s exact test)				
Complications	n (%)	n (% of 114)	n (% of 208)	p-value
GI mucosal injury (Zargar grade ≥IIa)†	41 (12.7%)	17 (14.9%)	24 (11.5%)	0.388
-corrosive-ingestion cases only (n=85)‡	41/85 (48.2%)	17/28 (60.7%)	24/57 (42.1%)	0.165
Aspiration pneumonia	14 (4.3%)	14 (12.3%)	0 (0.0%)	<0.001
Acute kidney injury (AKI)	14 (4.3%)	7 (6.1%)	7 (3.4%)	0.262
Arrhythmia	12 (3.7%)	8 (7.0%)	4 (1.9%)	0.030
Acute respiratory distress syndrome (ARDS)	7 (2.2%)	7 (6.1%)	0 (0.0%)	<0.001
Neurological deficit at discharge	8 (2.5%)	5 (4.4%)	3 (1.4%)	0.137
Laboratory parameters - Median (IQR) (Mann–Whitney U test, two-tailed)				
Laboratory parameters	Overall	Poor outcome	No poor outcome	p-value
Haemoglobin (g/dL)	12.4 (11.6–13.4)	12.4 (11.5–13.5)	12.5 (11.6–13.4)	0.713
Total leucocyte count (/µL)	9,395 (7,802–11,080)	10,071 (8,386–12,096)	9,153 (7,455–10,667)	<0.001
Platelet count (×10³/µL)	235 (190–272)	231 (180–281)	235 (192–268)	0.955
Random blood sugar (mg/dL)	144 (115–172)	151 (127–181)	136 (112–160)	<0.001
Urea (mg/dL)	38 (27–47)	40 (31–49)	36 (26–45)	0.008
Creatinine (mg/dL)	1.01 (0.75–1.34)	1.25 (0.86–1.56)	0.94 (0.72–1.22)	<0.001
pH§	7.36 (7.32–7.39)	7.33 (7.29–7.36)	7.37 (7.34–7.40)	<0.001
Bicarbonate (mEq/L)	21.8 (19.5–24.2)	20.3 (18.4–22.5)	22.7 (20.4–24.9)	<0.001
Sodium (mEq/L)	136.6 (134.1–139.0)	136.7 (134.4–139.4)	136.6 (134.1–138.8)	0.743
Potassium (mEq/L)	4.0 (3.7–4.3)	4.0 (3.7–4.4)	4.0 (3.7–4.3)	0.520
AST (U/L)	40 (19–68)	46 (24–71)	39 (18–64)	0.106
ALT (U/L)	36 (15–59)	41 (23–66)	34 (14–58)	0.062
PT/INR	1.05 (0.95–1.21)	1.06 (0.94–1.19)	1.05 (0.95–1.21)	0.659

Univariate and multivariable predictors of poor outcome

Using the composite poor outcome endpoint (ICU admission, mechanical ventilation, vasopressor support, or death; n=114, 35.4%; all 114 composite events included ICU admission), univariate analysis identified several significant predictors. Among categorical variables, SpO₂ <90% on admission had the largest computable univariate odds-ratio estimate (OR 15.16, 95% CI 5.70-40.34, p<0.001), followed by OP poisoning (OR 2.25, 95% CI 1.17-4.33, p=0.016). Shock on admission (n=41) and GCS <8 (n=27) were associated with poor outcome in all affected cases (Fisher's exact p<0.001 for both), precluding OR calculation due to complete separation. Male sex and alcohol co-ingestion were not significant predictors. Among continuous variables, lower GCS score (median 15 (IQR 8-15) in poor outcome vs 15 (IQR 15-15) in no poor outcome, p<0.001), lower SpO₂ (median 94%, IQR 89-96 vs 96%, IQR 94-98, p<0.001), lower systolic BP (104 vs 109 mmHg, p<0.001), higher HR (106 vs 93/min, p<0.001), higher creatinine (1.3 vs 0.9 mg/dL, p<0.001), and lower bicarbonate (20.3 vs 22.7 mEq/L, p<0.001) were all significantly associated with poor outcome.

**Figure 4:**
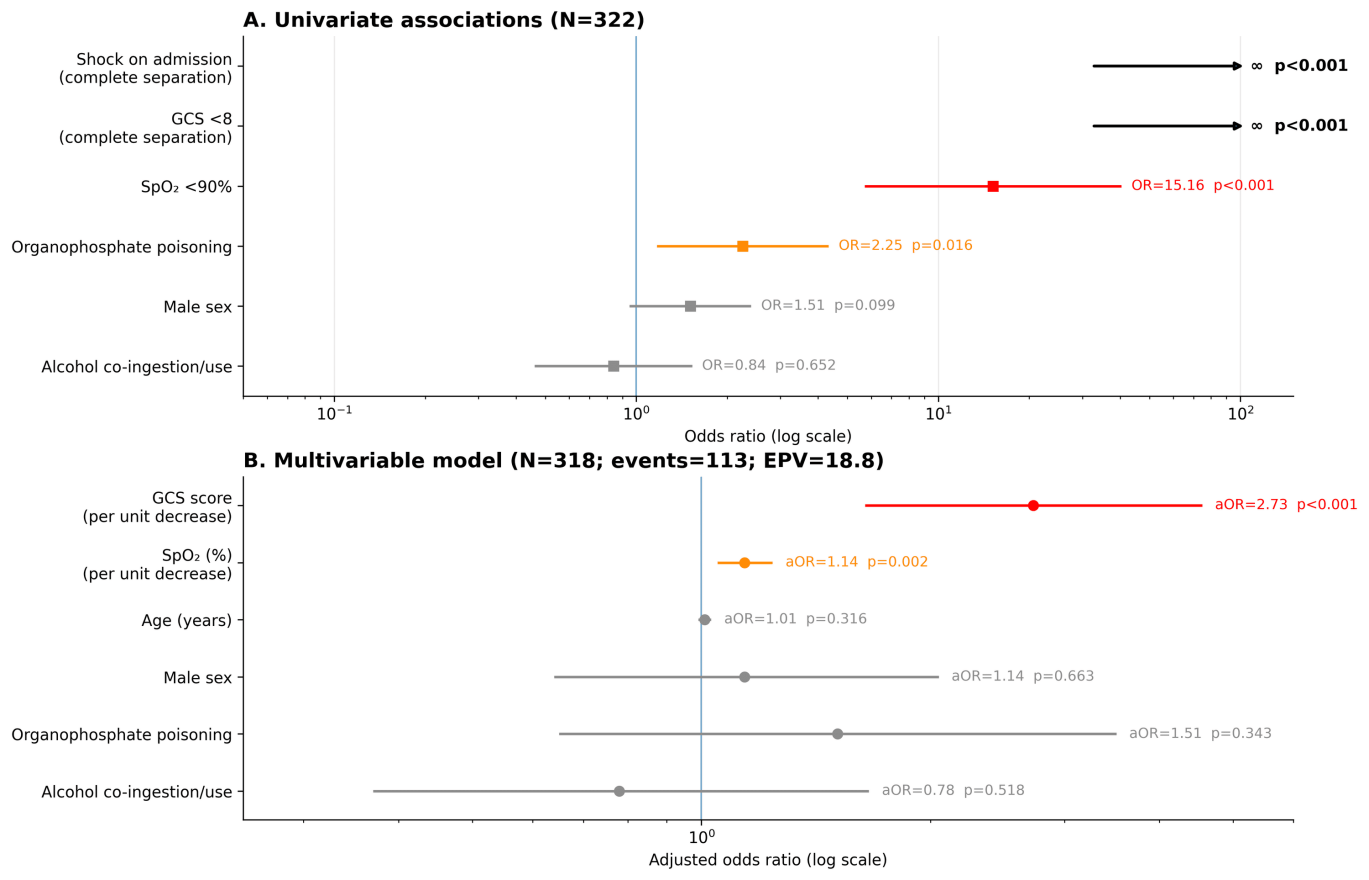
Forest Plot of univariate and multivariable associations with composite poor outcome (univariate N=322; multivariable N=318) Odds ratios (ORs) with 95% confidence intervals (CIs). The vertical reference line at OR=1.0 indicates no effect. Upper section — Univariate ORs (categorical predictors): GCS <8 and shock on admission demonstrated complete separation (all 27 GCS<8 patients and all 41 shocked patients met the composite endpoint); shown at OR=∞ and labelled accordingly. SpO₂ <90% had the largest computable univariate OR estimate (15.16, 95% CI 5.70–40.34; p<0.001). Colour coding: red = p<0.001; amber = p<0.05; grey = not significant. Lower section-Adjusted ORs from multivariable logistic regression (N=318, Events=113, EPV=18.8): GCS score (adjusted OR 2.73 per unit decrease, p<0.001) and SpO₂ (adjusted OR 1.14 per unit decrease, p=0.002) were independently associated with the composite endpoint. Hosmer-Lemeshow χ²=4.30 (df=8, p=0.829). OR = odds ratio; CI = confidence interval; GCS = Glasgow Coma Scale; SpO₂ = peripheral oxygen saturation; OP = organophosphate; EPV = events per variable.

On multivariable logistic regression (N=318, events=113, EPV=18.8), lower GCS score was independently associated with the composite outcome (adjusted OR 2.73 per unit decrease, 95% CI 1.64-4.55, p<0.001), as was lower SpO₂ (adjusted OR 1.14 per unit decrease, 95% CI 1.05-1.24, p=0.002). Age, sex, OP poisoning, and alcohol co-ingestion were not independently associated with the outcome after adjustment. Model Hosmer-Lemeshow test showed no evidence of lack of fit (χ²=4.30, df=8, p=0.829). McFadden pseudo-R²=0.275; overall accuracy=78.6%. The six-variable model demonstrated an apparent in-sample AUC of 0.765 (95% CI 0.712-0.824) and a Brier score of 0.156. At a predicted-probability threshold of 0.50, sensitivity was 46.0%, specificity 96.6%, positive predictive value 88.1%, and negative predictive value 76.4%. These are apparent in-sample estimates requiring external validation. Univariate predictors are presented in Table 7, multivariable logistic regression results in Table 8, and model-performance statistics in Table 9. The forest plot of univariate and multivariable associations is shown in Figure 4.

**Table 7:** Univariate predictors of composite poor outcome among patients with acute poisoning (N = 322) Poor outcome = ICU admission OR mechanical ventilation OR vasopressor support OR in-hospital death (n=114, 35.4%). For univariate categorical predictors, n (row %) = proportion within the exposed group, not proportion of the 114 poor-outcome events (e.g., 56 [40.9%] for male sex = 56 of 137 males had a poor outcome). † Complete separation: all 27 GCS <8 cases and all 41 shocked patients met the composite endpoint; OR is undefined. ‡ Shock was not entered into the multivariable model because of complete separation (Fisher's exact p<0.001). OR = odds ratio; CI = confidence interval; GCS = Glasgow Coma Scale; SpO₂ = peripheral oxygen saturation; OP = organophosphate; ICU = intensive care unit; IQR = interquartile range; ns = not significant; *** p<0.001; ** p<0.01; * p<0.05.

Variable	Poor Outcome n (row %)	No Poor Outcome n (row %)	Crude OR (95% CI)	p-value
Categorical Variables (Fisher's Exact Test)				
GCS <8 (n=27)	27 (100.0)	0 (0.0)	Complete separation†	<0.001***
Shock on admission (n=41)	41 (100.0)	0 (0.0)	Complete separation†	<0.001***
SpO₂ <90% (n=36)	31 (86.1)	5 (13.9)	15.16 (5.70–40.34)	<0.001***
Organophosphate poisoning (n=42)	22 (52.4)	20 (47.6)	2.25 (1.17–4.33)	0.016*
Male sex (n=137)	56 (40.9)	81 (59.1)	1.51 (0.95–2.40)	0.099
Alcohol co-ingestion (n=59)	19 (32.2)	40 (67.8)	0.84 (0.46–1.53)	0.652
Continuous Variables, Median (IQR) (Mann–Whitney U Test)				
GCS score	15 (8–15)	15 (15–15)	—	<0.001***
SpO₂ (%)	94 (89–96)	96 (94–98)	—	<0.001***
Systolic blood pressure (mmHg)	104 (90–112)	109 (100–118)	—	<0.001***
Heart rate (/min)	106 (94–117)	93 (83–103)	—	<0.001***
Creatinine (mg/dL)	1.3 (0.9–1.6)	0.9 (0.7–1.2)	—	<0.001***
Bicarbonate (mEq/L)	20.3 (18.4–22.5)	22.7 (20.4–24.9)	—	<0.001***
Age (years)	32 (22–41)	28 (21–37)	—	0.102

**Table 8:** Multivariable logistic regression-variables independently associated with composite poor outcome (N=318). Adjusted OR = odds ratio adjusted for all model variables; CI = confidence interval; EPV = events per variable; VIF = variance inflation factor; GCS = Glasgow Coma Scale; SpO₂ = peripheral oxygen saturation; OP = organophosphate. GCS and SpO₂ adjusted ORs are reported per unit decrease. EPV=18.8.

Variable	Adjusted OR	95% Confidence Interval	p-value	Significance
GCS score (per unit decrease)	2.73	1.64–4.55	<0.001	***
SpO₂ (%) (per unit decrease)	1.14	1.05–1.24	0.002	**
Age (years)	1.01	0.99–1.03	0.316	ns
Male sex	1.14	0.64–2.05	0.663	ns
Organophosphate poisoning	1.51	0.65–3.51	0.343	ns
Alcohol co-ingestion	0.78	0.37–1.66	0.518	ns
Shock on admission	Not entered‡	-	-	-

**Table 9:** Model performance statistics-multivariable logistic regression (N=318) Apparent in-sample performance: AUC 0.765 (95% CI 0.712–0.824), Brier score 0.156; at a probability threshold of 0.50, sensitivity 46.0%, specificity 96.6%, positive predictive value 88.1%, negative predictive value 76.4%, and overall classification accuracy 78.6%. Hosmer–Lemeshow χ²=4.30, df=8, p=0.829; McFadden pseudo-R²=0.275. These estimates require internal and external validation. AUC = area under the receiver operating characteristic curve; HL = Hosmer–Lemeshow.

Parameter	Value
Study population included (N)	318
Composite outcome events (n)	113
Events per variable (EPV)	18.8
Apparent model AUC	0.765 (95% bootstrap CI 0.712–0.824)
Brier score	0.156
Probability threshold	0.50
Sensitivity	46.0%
Specificity	96.6%
Positive predictive value	88.1%
Negative predictive value	76.4%
Overall classification accuracy	78.6%
Hosmer–Lemeshow χ² (df=8)	4.30
Hosmer–Lemeshow p-value	0.829 (no evidence of lack of fit)
McFadden pseudo-R²	0.275
Variance inflation factors (VIF)	
GCS score	1.37
SpO₂ (%)	1.36
Age (years)	1.07
Male sex	1.07
Organophosphate poisoning	1.04
Alcohol co-ingestion/use	1.02

ROC analysis

**Figure 5:**
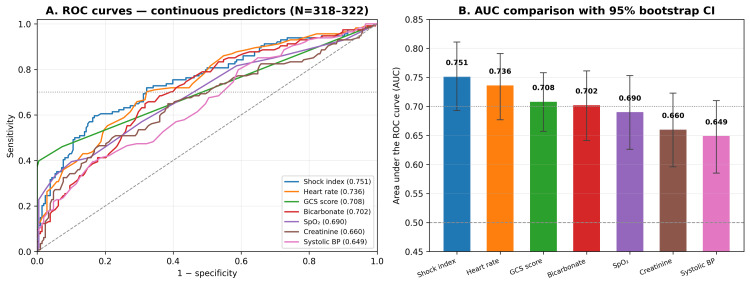
ROC curves and AUC comparison for continuous predictors of composite poor outcome (N=318–322, depending on predictor) (A) ROC curves for seven continuous predictors plotted as sensitivity against 1-specificity. The diagonal dashed line in Panel A indicates no discrimination. In Panel B, the dotted horizontal line indicates AUC=0.70 and the dashed horizontal line indicates AUC=0.50. The shock index (HR/SBP) curve is highlighted (AUC 0.751). (B) Bar chart comparing AUC values with 95% bootstrap CI (1,000 iterations, seed=42). AUC values (descending): shock index 0.751 (0.693–0.811); heart rate 0.736 (0.677–0.791); GCS score 0.708 (0.657–0.758); bicarbonate 0.702 (0.641–0.761); SpO₂ 0.690 (0.626–0.753); creatinine 0.660 (0.596–0.723); systolic BP 0.649 (0.585–0.710). Optimal Youden-index cutpoints: shock index ≥1.01 (Se=0.60, Sp=0.83); HR ≥99/min (Se=0.70, Sp=0.68); GCS ≤13 (Se=0.40, Sp=1.00). All cutpoints are data-derived and require external prospective validation. AUC = area under the ROC curve; HR = heart rate; SBP = systolic blood pressure; GCS = Glasgow coma scale; SpO₂ = peripheral oxygen saturation; Se = sensitivity; Sp = specificity; CI = confidence interval.

The shock index (HR/SBP ratio) was pre-specified for exploratory ROC evaluation as a potential bedside severity marker. In the overall cohort, the median shock index was 0.91 (IQR 0.77-1.07); it was higher among patients meeting the composite endpoint (median 1.07, IQR 0.90-1.25) than among those who did not (median 0.85, IQR 0.74-0.97). The shock index had the largest point-estimate AUC (0.751, 95% CI 0.693-0.811), followed by heart rate (AUC 0.736, 95% CI 0.677-0.791), GCS score (AUC 0.708, 95% CI 0.657-0.758), bicarbonate (AUC 0.702, 95% CI 0.641-0.761), SpO₂ (AUC 0.690, 95% CI 0.626-0.753), creatinine (AUC 0.660, 95% CI 0.596-0.723), and systolic BP (AUC 0.649, 95% CI 0.585-0.710). Confidence intervals overlapped; however, no formal pairwise comparison of AUCs was performed, and statistical equivalence should not be inferred from overlapping intervals alone. The optimal GCS cutoff was ≤13 (sensitivity 0.40, specificity 1.00), and the optimal SpO₂ cutoff was ≤91% (sensitivity 0.39, specificity 0.90). All cutpoints are data-derived and require external prospective validation before clinical application as decision thresholds. The ROC findings are summarised in Table 10 and illustrated in Figure 5.

**Table 10:** ROC analysis-discriminatory value of continuous predictors for composite poor outcome (N=318–322, depending on predictor) AUC = area under the ROC curve (range 0–1; 1.0 = perfect discrimination, 0.5 = no discrimination). 95% CI derived from 1,000 bootstrap iterations (seed=42). Optimal cutpoints determined by the Youden index (maximising sensitivity + specificity − 1). GCS, SpO₂, bicarbonate, and SBP were inverted for ROC analysis (lower value = higher risk). Sensitivity = proportion of poor-outcome cases correctly identified; Specificity = proportion of no-poor-outcome cases correctly identified. All cutpoints require external prospective validation before clinical application. AUC interpretation: ≥0.9 excellent; 0.8–0.89 good; 0.7–0.79 acceptable; <0.7 poor. HR = heart rate; SBP = systolic blood pressure; GCS = Glasgow coma scale; SpO₂ = peripheral oxygen saturation.

Predictor	AUC	95% CI (bootstrap)	Optimal cutoff (Youden index)	Sensitivity	Specificity
Shock index (HR/SBP)	0.751	0.693–0.811	≥ 1.01	0.60	0.83
Heart rate (/min)	0.736	0.677–0.791	≥ 99	0.70	0.68
GCS score	0.708	0.657–0.758	≤ 13	0.40	1.00
Bicarbonate (mEq/L)	0.702	0.641–0.761	≤ 21.2	0.65	0.68
SpO₂ (%)	0.690	0.626–0.753	≤ 91%	0.39	0.90
Creatinine (mg/dL)	0.660	0.596–0.723	≥ 1.3	0.46	0.83
Systolic BP (mmHg)	0.649	0.585–0.710	≤ 95	0.39	0.84

Outcomes and mortality

**Figure 6:**
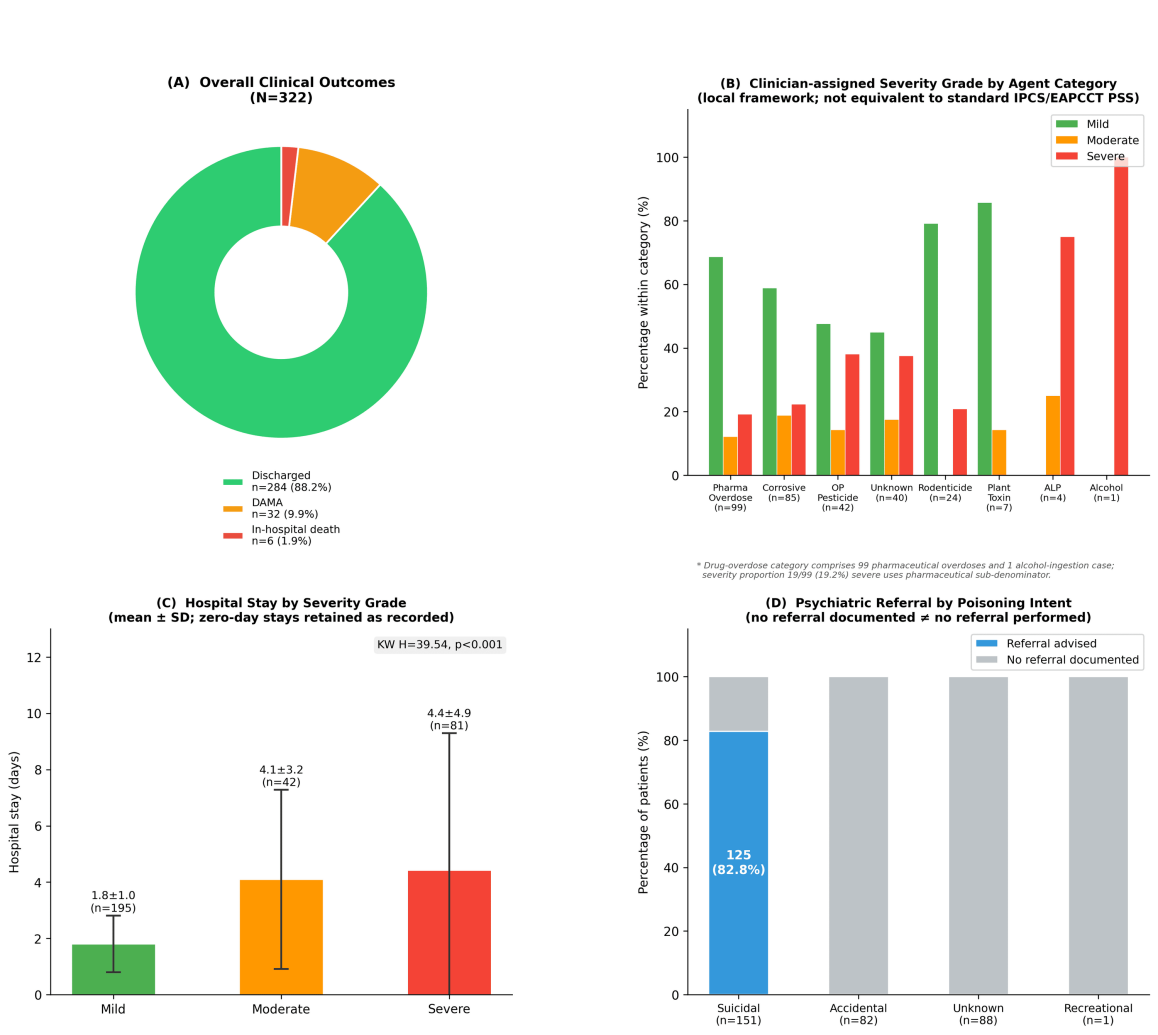
Overall clinical outcomes, severity grade by agent category, hospital stay distribution, and psychiatric referral pattern (N=322) (A) Donut chart showing overall clinical outcomes: Discharged n=284 (88.2%); DAMA n=32 (9.9%); In-hospital death n=6 (1.9%). Mortality among patients with a documented discharge or death outcome was 2.1% (6/290). Post-admission mortality may have been underestimated because outcomes after DAMA were unavailable. (B) Grouped bar chart showing proportions of Mild/Moderate/Severe cases within each major agent category. ALP showed the highest severe-grade proportion (3/4, 75%), followed by OP (16/42, 38.1%) and unknown agent (15/40, 37.5%). Pharmaceutical overdose (n=99 of the 100-patient drug-overdose category) had a severe proportion of 19/99 (19.2%); the alcohol-ingestion case included in the drug-overdose category was not severe. (C) Bar chart with error bars (±1 SD) showing mean hospital stay by severity grade: Mild 1.8 ± 1.0 days (median 2); Moderate 4.1 ± 3.2 days (median 3); Severe 4.4 ± 4.9 days (median 3); Overall 2.8 ± 3.1 days (n=318). Mean ICU stay = 3.4 days (n=114). (D) Bar chart showing psychiatric referral rates by poisoning intent: suicidal cases n=125/151 (82.8%); accidental and unknown-intent cases: 0 referrals documented. DAMA = discharge against medical advice; ALP = aluminium phosphide; OP = organophosphate; ICU = intensive care unit; SD = standard deviation.

Among the 322 cases, 284 were discharged following treatment (88.2%), 32 left against medical advice (DAMA, 9.9%), and six died, yielding an overall in-hospital mortality of 1.9% (6/322). Among the 290 patients with a documented discharge or death outcome, mortality was 2.1% (6/290). Mean hospital stay was 1.8 days for mild-grade, 4.1 days for moderate-grade, and 4.4 days for severe-grade cases. The relatively short mean stay for severe cases may partly reflect early deaths (fatal-case stays ranged from one day for the three ALP deaths to five and seven days for one corrosive and one OP death; hospital-stay data were unavailable for one fatal corrosive case) and DAMA, which occurred after a median hospital stay of one day, rather than rapid clinical recovery. A subset of severe corrosive and OP cases had prolonged stays of up to 21 and 26 days, respectively. Three of the four patients with ALP poisoning died. Among patients with a documented discharge or death outcome, mortality was 2.7% for corrosive poisoning (2/74) and 2.5% for OP poisoning (1/40); the corresponding crude in-hospital mortality proportions using the complete category denominators were 2.4% each. No in-hospital death occurred in the drug-overdose category. Five of the six deaths occurred in male patients, although the small number of deaths precluded reliable inferential analysis, and males constituted 42.5% of admissions. Outcomes by agent category and the clinical profile of all fatal cases are presented in Tables 11 and 12. The overall clinical outcome distribution, severity by agent category, hospital stay, and psychiatric referral patterns are illustrated in Figure 6.

**Table 11:** Clinical outcomes according to poisoning agent category (N = 322) †The drug-overdose category (n=100) comprises 99 pharmaceutical-overdose cases and one alcohol-ingestion case; the combined outcomes were 91 discharges, nine DAMA cases and no in-hospital deaths.*Mortality among patients with a documented discharge or death outcome = deaths / (deaths + discharges) × 100%; patients discharged against medical advice (DAMA) were excluded from this denominator because post-discharge outcomes were unavailable. Crude in-hospital mortality uses the full agent-category denominator. DAMA = discharge against medical advice; ALP = aluminium phosphide; OP = organophosphate.

Agent Category	N	Discharged n (%)	DAMA n (%)	In-hospital death n (%)	Mortality among documented outcomes* (%)	Mean Stay (days)
Drug overdose†	100	91 (91.0%)	9 (9.0%)	0 (0.0%)	0.0	2.1
Corrosive ingestion	85	72 (84.7%)	11 (12.9%)	2 (2.4%)	2.7	3.1
Organophosphate poisoning	42	39 (92.9%)	2 (4.8%)	1 (2.4%)	2.5	5.0
Unknown agent	40	36 (90.0%)	4 (10.0%)	0 (0.0%)	0.0	2.5
Rodenticide poisoning	24	21 (87.5%)	3 (12.5%)	0 (0.0%)	0.0	2.0
Other insecticide poisoning	20	18 (90.0%)	2 (10.0%)	0 (0.0%)	0.0	2.1
Plant toxin poisoning	7	6 (85.7%)	1 (14.3%)	0 (0.0%)	0.0	1.9
Aluminium phosphide poisoning	4	1 (25.0%)	0 (0.0%)	3 (75.0%)	75.0	1.5
Total	322	284 (88.2%)	32 (9.9%)	6 (1.9%)	2.1	2.8

**Table 12:** Clinical characteristics and course of all in-hospital fatal cases (n = 6) GCS = Glasgow coma scale score at admission; ICU = intensive care unit; AKI = acute kidney injury; ARDS = acute respiratory distress syndrome. All six fatal cases were classified as severe, admitted to the ICU, and mechanically ventilated. Only information recorded in the final analytic dataset is shown.

Age	Sex	Category	Agent	GCS	Ventilation	Vasopressor	Recorded complication(s)	Stay (days)
65	Female	Corrosive ingestion	Not recorded	7	Yes	Yes	Aspiration pneumonia, GI injury	5
52	Male	Organophosphate	unknown	6	Yes	No	No complication documented in CRF	7
17	Male	Aluminium phosphide	Aluminium Phosphide	6	Yes	Yes	AKI	1
40	Male	Aluminium phosphide	Aluminium Phosphide	5	Yes	Yes	No complication documented in CRF	1
19	Male	Aluminium phosphide	Aluminium Phosphide	5	Yes	Yes	ARDS, Arrhythmia	1
70	Male	Corrosive ingestion	Lysol	15	Yes	Yes	No complication documented in CRF	Not recorded

## Discussion

This study presents a combined descriptive and analytical evaluation of acute poisoning at a tertiary care hospital serving an industrial-urban catchment in eastern India. Pharmaceutical overdose and household chemical ingestion together accounted for 57.5% of admissions, alongside OP pesticide, rodenticide, plant-toxin, and occupational chemical exposures. Industrial and occupational cases constituted 3.4% of the cohort and are presented as a descriptive local feature. The analytical findings identify admission variables associated with an institution-dependent escalation-of-care endpoint and should be interpreted as exploratory.

The industrial-urban poisoning epidemiology

Young adults formed the largest age group, and suicidal intent was documented in 46.9% of admissions. Although occupational and socioeconomic stressors may be relevant in industrial populations, these constructs were not measured in this study, and no causal attribution can be made. Alcohol co-ingestion or habitual use was documented in 18.3% of patients, but was not independently associated with the composite endpoint.

The female predominance (56.5%) warrants particular attention. This finding is consistent with South Asian data showing that impulsive self-poisoning by young women, often in the context of acute psychosocial stressors, is a primary driver of poisoning admissions even in cities where men constitute the majority of the formal workforce [11,12]. The present study did not collect data on specific precipitating circumstances. The accessibility of toxic household agents may represent a relevant area for future prevention research [13]. The history of prior self-harm in 11.8% reinforces the need for structured post-discharge follow-up.

Eleven cases (3.4%) involved industrial chemicals or occupational exposures, an infrequently described category in the Indian tertiary-care poisoning series. These were distributed across corrosive, unknown agent, and other insecticide categories based on the substance involved; formal toxicological confirmation was not universally available, and diagnoses were based on clinical presentation and exposure history. The small number precludes category-specific conclusions, but underscores the distinct occupational-exposure dimension of this industrial catchment [10,14].

Dual burden: urban pharmaceuticals and regional pesticides

In this hospital cohort, pharmaceutical overdose (31.1%) and household chemical ingestion (26.4%) were recorded more frequently than OP pesticide poisoning (13.0%). The observed agent distribution may inform the design of future prospective studies and local preparedness assessments, but it does not establish a temporal epidemiological transition. Drug overdose accounted for 31.1% of admissions compared with 13.0% for OP poisoning. Benzodiazepines and related hypnotics were the largest pharmaceutical subclass (n=40), with alprazolam recorded in 14 cases; no in-hospital death occurred in the drug-overdose category [7,15]. Among drug-overdose cases, ECG findings included sinus tachycardia (n=21), arrhythmia (n=5), QT prolongation (n=2), and ST-T changes (n=1); the specific agents responsible were not uniformly documented in the CRF.

Corrosive ingestion (26.4%), overwhelmingly involving phenyl-based household floor cleaners (72.9%, n=62), is a characteristic feature of urban Indian poisoning series. GI injury (Zargar ≥IIa) occurred in 48.2% of corrosive cases (41 of 85), predominantly grade IIa-IIb ulceration [16]. Upper gastrointestinal endoscopy was performed in 58 patients (68.2% of corrosive cases), all of whom had corrosive ingestion; no endoscopies were performed in other agent categories. Long-term consequences, including oesophageal stricture, were not captured in this retrospective dataset. Paracetamol overdose (n=20) was managed without fatality in this series; N-acetylcysteine should remain available according to established protocols [17].

OP poisoning (13.0% of admissions) had an ICU admission rate of 52% (22/42), the highest among major agent categories, and required substantial ventilatory and antidote resources. Atropine was administered in 92.9% (39/42) and pralidoxime in 66.7% (28/42) of OP cases. Patients with clinically significant OP poisoning may require continued monitoring beyond initial atropinisation because delayed respiratory deterioration can occur [18,19]. Serum cholinesterase was available in all 42 OP cases but was not analysed across the full cohort as it was not routinely measured in non-OP categories.

Severity, vital signs, and the poor outcome burden

A composite poor outcome was identified in 114 patients (35.4%). The low overall crude in-hospital mortality (1.9%; 6/322) alongside a 35.4% ICU admission rate reflects the institution's low-threshold combined ICU-HDU monitoring policy. In this cohort, all 114 patients meeting the composite endpoint were admitted to the ICU; no patient met the ventilation, vasopressor, or mortality component without also receiving ICU admission. The composite endpoint is therefore numerically equivalent to ICU admission in this cohort and should be interpreted accordingly; it reflects both clinical severity and institutional ICU-triage thresholds, and may not be directly comparable across centres with different escalation criteria. ICU mortality was 5.3% (6/114). Drug overdose and corrosive ingestion together constituted 57.5% of admissions but accounted for relatively lower severity, consistent with their predominantly mild clinical severity, although severe cases occurred in both categories.

The finding that 28.9% of admissions presented with SpO₂ below 94% and 11.2% below 90% at triage reflects the high proportion of OP poisoning, severe drug overdose, and corrosive-associated aspiration in this cohort. SpO₂ below 90% had one of the largest computable univariate odds-ratio estimates for the escalation-of-care endpoint (OR 15.16, 95% CI 5.70-40.34 for SpO₂ <90%, p<0.001) and was independently associated with the escalation-of-care endpoint on multivariable regression (adjusted OR 1.14 per unit decrease, p=0.002). These findings reinforce the use of pulse oximetry and respiratory assessment as potentially useful bedside triage parameters in resource-limited acute poisoning settings [20].

The shock index had the largest point-estimate AUC among the evaluated continuous variables (0.751, 95% CI 0.693-0.811), followed by heart rate and GCS. Their confidence intervals overlapped; however, no formal pairwise comparison of AUCs was performed, and statistical equivalence should not be inferred from overlapping intervals alone. Its bedside simplicity supports prospective evaluation as a potential severity marker; the data-derived cutpoint of ≥1.01 requires external validation before clinical application [21-23]. Shock was recorded in 41 patients, all of whom were admitted to the ICU; 32 (78.0%) received vasopressor support. Because ICU admission was part of the composite endpoint, the complete association between shock and the endpoint is affected by incorporation bias and should be interpreted primarily as reflecting institutional escalation practice. Alcohol co-ingestion or habitual use was documented in 18.3% of patients and was not significantly associated with the composite endpoint in univariate analysis (OR 0.84, 95% CI 0.46-1.53, p=0.652) or after multivariable adjustment (adjusted OR 0.78, 95% CI 0.37-1.66, p=0.518). The direction of the association is not formally explained by this study.

Complications: agent-specific patterns

Aspiration pneumonia was documented in 14 patients (all met the composite endpoint; poor=14, not poor=0; p<0.001), distributed across the following categories: unknown-agent (n=5), drug-overdose (n=3), OP (n=2), corrosive (n=2), and others. Arrhythmia was documented in 12 patients (poor=8, not poor=4; p=0.030). ARDS occurred in seven patients (all met the composite endpoint; p<0.001). Gastrointestinal mucosal injury was documented in 41 patients, including 17 of 114 patients meeting the composite endpoint and 24 of 208 patients not meeting the endpoint (14.9% versus 11.5%; Fisher’s exact p=0.388). The retrospective data were not designed to evaluate specific management protocols. Acute kidney injury (AKI) was documented in 14 patients (4.3%), most frequently in the drug-overdose category (n=7), with smaller numbers in corrosive (n=2), OP (n=2), and other categories; none required dialysis. The study did not evaluate a specific renal-monitoring strategy.

Gastric lavage was performed in 45.0% of patients. Contemporary guidance does not support routine lavage and recommends that any exceptional use be undertaken only by appropriately trained clinicians after careful consideration of timing, substance, airway protection, and contraindications [24]. The available retrospective analysis did not evaluate lavage according to exact agent, time from exposure, airway protection, or contraindications, and therefore cannot establish the appropriateness of individual decisions.

Aluminium phosphide: a high-lethality threat

ALP constituted only 1.2% of cases. Three of the four patients died, three required mechanical ventilation and vasopressor support, and all four were admitted to the ICU. This descriptive finding is based on a very small subgroup and carries substantial statistical imprecision; it should be interpreted as a clinical signal rather than a stable epidemiological estimate [25].

Psychiatric dimensions and the DAMA challenge

Psychiatric referral was advised in 82.8% of suicidal poisoning admissions (n=125/151), reflecting systematic recognition of the mental health dimension of intentional self-poisoning. Direct comparison with other studies is difficult because referral practices, eligibility criteria, and reporting methods vary [26,27]. Notably, no psychiatric referral was documented among accidental or unknown-intent admissions (n=171); absence of documentation does not confirm that no referral was clinically performed. This represents an area for prospective evaluation of structured psychosocial screening [13].

The 9.9% DAMA rate represents a persistent structural challenge. Prospective studies could evaluate whether early psychiatric assessment and structured family engagement reduce DAMA among patients with intentional poisoning. Whether some cases classified as accidental or unknown intent reflect undisclosed suicidal ideation is a hypothesis not evaluable in the present study and warrants prospective investigation with structured intent-assessment instruments [13,26].

Comparison with other Indian and regional series

The overall in-hospital mortality of 1.9% is consistent with published urban Indian tertiary-care series (range 1.0-4.5%) and lower than rates from rural agricultural series where OP and ALP predominate [8,9,28]. The relatively low in-hospital mortality may partly relate to the distribution of recorded agents, predominantly pharmaceutical overdoses and corrosive ingestion, the inclusion of mild-severity cases (60.9% of the cohort), early presentation in many patients (mean 3.2 hours), and institutional monitoring practices; causal explanations cannot be established from this retrospective study. Additionally, 32 patients (9.9%) were discharged against medical advice before outcome could be determined; some may have died after discharge, and post-admission mortality may have been underestimated because outcomes after DAMA were unavailable. The corrosive poisoning proportion (26.4%), the presence of an industrial chemical category, the benzodiazepine-dominant pharmaceutical profile, and the female predominance collectively distinguish this series from both agricultural and purely metropolitan Indian cohorts. Gastric lavage was performed in 45.0% of cases; interpretation of this rate requires time-to-presentation stratification by agent, although presentation time was recorded, the relationship between agent-specific presentation timing and gastric lavage appropriateness was not analysed in this study [24]. Pharmacological management of the OP intermediate syndrome warrants awareness in settings with high OP burden [29]. ECG monitoring should follow established toxicology protocols for drug overdoses associated with conduction abnormalities; the agents responsible for the observed QT prolongation and arrhythmia events were not uniformly documented in this cohort [30].

Limitations

First, complete laboratory availability reflects the centre's mandatory admission-workup protocol: serum cholinesterase was available in all 42 OP cases, but was not included in full-cohort analyses as it was not routinely measured across all categories. pH and bicarbonate were available for all patients; however, the specimen source (arterial, venous, or capillary) was not consistently documented in the CRF and cannot be confirmed retrospectively. Lactate and the complete arterial blood gas panel were not consistently captured, limiting detailed metabolic characterisation, particularly in ALP cases. Second, four patients with missing GCS scores were excluded from the multivariable regression model; one had experienced the composite outcome. Although the number was small, complete-case analysis may have introduced limited selection bias. Third, the single-centre design limits generalisability; a multi-centre prospective study across this industrial belt would substantially strengthen the regional evidence base. Fourth, complete separation was observed for shock and GCS <8: all 41 shocked patients and all 27 patients with GCS <8 met the composite poor outcome definition (100% in both groups). In this cohort, these features were closely linked to institutional escalation decisions, particularly ICU admission and, where clinically indicated, organ-support interventions, rather than reflecting biological unpredictability. These variables are more appropriately interpreted as markers of institutional escalation than as independent biological predictors. Fifth, severity was recorded using local clinician-assigned mild, moderate, and severe categories and may not have been applied uniformly throughout the study period. These categories were not equivalent to the standard IPCS/EAPCCT Poisoning Severity Score. Because the categories incorporated physiological derangement and organ-support requirements, comparisons across severity groups are partly circular and should be interpreted descriptively. Sixth, because ICU admission and organ-support interventions formed part of the composite outcome endpoint, some predictors may partially reflect clinician-driven escalation pathways rather than purely biological deterioration (incorporation bias); this should be considered when applying these findings elsewhere. Seventh, as a tertiary care centre, this hospital likely receives disproportionately severe cases transferred from peripheral facilities, potentially inflating severity and poor outcome rates. Eighth, DAMA patients (n=32, 9.9%) were lost to follow-up; delayed mortality after discharge cannot be captured, and post-admission mortality may have been underestimated. Ninth, retrospective CRF documentation introduces ascertainment bias, and toxicological confirmation by laboratory assay was not available for pharmaceutical, corrosive, or unknown-agent cases. Tenth, occupation, socioeconomic status, and formal stress assessment were not captured. Eleventh, penalised (Firth) logistic regression was not employed; this approach would have permitted effect size estimation for complete-separation variables (shock on admission, GCS <8), and is acknowledged as a methodological alternative. Twelfth, variable selection was guided by univariate screening and a priori clinical reasoning rather than a pre-registered analysis plan, which may have introduced selection bias. Thirteenth, the severity-grade framework partially incorporates vital sign derangement in its definition; Kruskal-Wallis comparisons of vital signs across severity grades are therefore partly circular and should be interpreted with this in mind. Fourteenth, subgroup analyses for aluminium phosphide (n=4) carry extreme statistical imprecision; mortality and severity estimates have very wide uncertainty intervals and should not be used for quantitative inference. Fifteenth, the multivariable model was developed and evaluated in the same cohort without optimism-corrected internal validation; the apparent AUC, sensitivity, specificity, and predictive values may overestimate performance in new patients, and both internal and external validation are required before clinical application.

## Conclusions

This study characterises the clinical and epidemiological profile of acute poisoning at a tertiary care hospital in the industrial urban setting of eastern India. The most frequently recorded categories in this single-centre cohort were pharmaceutical overdose and household chemical ingestion, alongside OP pesticide, rodenticide and occupational chemical exposures. Corrosive poisoning was associated with frequent endoscopically documented GI mucosal injury among scoped patients. Three of the four patients with ALP poisoning died; this descriptive finding was based on a very small subgroup. A substantial proportion of admissions (35.4%) met the composite poor outcome endpoint. Because all composite events in this cohort included ICU admission, the endpoint reflects both clinical severity and institutional ICU-triage practices and may not be directly comparable across centres with different escalation thresholds.

On multivariable logistic regression, lower admission GCS and SpO₂ were independently associated with the escalation-of-care endpoint, which was numerically equivalent to ICU admission in this cohort. The shock index had the largest point-estimate AUC among the evaluated continuous variables (0.751). No formal pairwise comparison of AUCs was performed. These exploratory associations require optimism-corrected internal validation and external validation using objective clinical outcomes before clinical application. The observed exposure distribution and management patterns may inform the design of prospective multicentre studies. Future research should use objective clinical outcomes, reproducible exposure and severity classifications, and validated analytical methods before making practice recommendations.
